# Minimally invasive approach in radio distal end fractures, three different types of incisions

**DOI:** 10.1093/jscr/rjab203

**Published:** 2021-05-27

**Authors:** Fernando Xavier Romero Prieto, Carlos Raúl Reyes García

**Affiliations:** Orthopaedy and Traumatology Department, Microsurgery Unit, Roosevelt Hospital, Guatemala City, Guatemala; Orthopaedy and Traumatology Department, San Carlos University, Roosevelt Hospital, Guatemala City, Guatemala

## Abstract

Two techniques are described to treat distal radius fractures: conventional (Henry approach) and minimally invasive plate osteosynthesis. The latter technique has been described by different authors such as Imatani *et al*. and Zenke *et al*.

This was a descriptive retrospective study, analyzing 26 adult patients with unstable distal radius fracture, extra-articular type A or partially intra-articular type B according to AO. The approaches used were: (i) single longitudinal palmar incision; (ii) double T-incision (horizontal and vertical) and (iii) double position II.

Ages were between 21 and 78 years. Most affected hand was the right. Most common fracture was 23B2 (AO classification). In total, 84.6% of the patients did not present complications. According to the functional evaluation of the wrist by the Mayo Clinic, 31% showed excellent results, 42% showed good results, 27% showed fair results.

The techniques had satisfactory results for the osteosynthesis with more aesthetic and less invasive approach.

## INTRODUCTION

When we talk about surgical treatment, the first three things that come to the mind of the patient are recovery time, how big the surgery will be and aesthetics (scar) [1]. Being distal radius fractures the second most common fracture in the locomotor system [2], the need to treat distal radius fractures surgically has increased because nonsurgical treatment frequently delays the patient’s return to the activities of daily living [3, 4]. Hence, there is a need to describe new approaches, more aesthetic and less invasive with the same effectiveness as the conventional method for greater patient comfort [3]. The objective of the article is to describe three new approaches for the osteosynthesis of distal radius fractures with locked plates of variable angle. The techniques are minimally invasive, preserving the pronator square muscle [5], making a minimal incision, which would make the early return to regular activities.

## CASE REPORT/SERIES

The study was a retrospective review of 26 patients from the Hand Unit of the Traumatology and Orthopedics Department of the authors’ affiliated institutions. Once the procedure was approved by the institutional review board of the authors’ affiliated institutions, patients signed an informed consent for the use of photos and videos of their wrists and the radiographs used in this publication. The patients were selected in the Hospital Emergency Room by the Department of Traumatology and Orthopedics. The exclusion criteria were patients who presented exposed fractures or simple radial fractures with long segment metadiaphyseal comminution and neurovascular injuries of the forearm or polytraumatized patients. All fractures were classified according to AO rules, five were 23-A2; five were 23-A3; five were 23-B1; six were 23B-2; two were 23-B3 and three were 23-C1. The average time between the accident and the surgery was 7.3 ± 2.7 days, (between 5 and 14 days).

### Surgical technique

The patient was placed on an operating table in a supine position. Under general anesthesia, the surgeon and assistant tractioned the patient’s forearm longitudinally, with the forearm in full pronation and the elbow extended.

With this traction, the surgeon performed a manual reduction. Once a satisfactory reduction under radiographic control was achieved, surgeon inserted one or two high-res 1.5-mm Kirschner wires obliquely to maintain reduction temporarily, followed by placing the forearm in full supination. Three types of incision were made.

#### T Orientation

**Figure 1:**
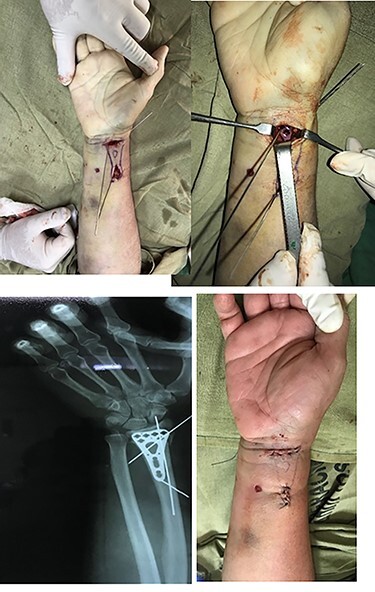
T shape incision. (**a**) The forearm with the two incisions. (**b**) Visualizing the plate. (**c**) X-ray visualizing the correct position of the plate. (**d**) Both incisions sutured.

First, we start with a 2.5-cm long transverse orientation incision in the proximal wrist crease to locate the brachial fascia and flexor carpi radialis (FCR; Fig. 1). An additional dissection was performed in the interval between the FCR laterally and the radial artery medially. Tendon of the flexor pollicis longus is retracted medially to expose the pronator quadratus (PQ), which in this incision is respected (<1.5 cm) to expose the anterior cortex of the distal radius and observe the direct reduction the fracture. The extraperiosteal tunnel was made between the PQ muscle and the periosteum of the radius. A 2.4-mm titanium variable angle-locking plate was inserted through the transverse incision, slid through the fracture site and reduced and aligned on the radius through the longitudinal incision in the forearm.

A 1.5-mm Kirschner wire was then inserted through a distal screw drill guide into the plate to temporarily fix the plate. The alignment, length and rotation of the fracture were controlled openly, and any obvious deformity was corrected by manual manipulation. Once an anatomical alignment is achieved, radial vessels were laterally retracted, whereas the FCR retracted medially and then inserted another 1.5-mm wire through a guide proximal drill on plate.

Two suitable locking screws were placed through the distal and proximal incisions. The two anterior Kirschner wires were then removed, and other locking screws were placed in the distal segment, taking care not to place the screws in the wrist joint. The screws were then placed at the proximal end of the plate. The incisions were closed in planes, and only dressing with sterile gauze over the wounds was done and an elastic bandage was used in order to initiate early mobility.

##### Longitudinal single incision I

###### Orientation of incision

**Figure 2:**
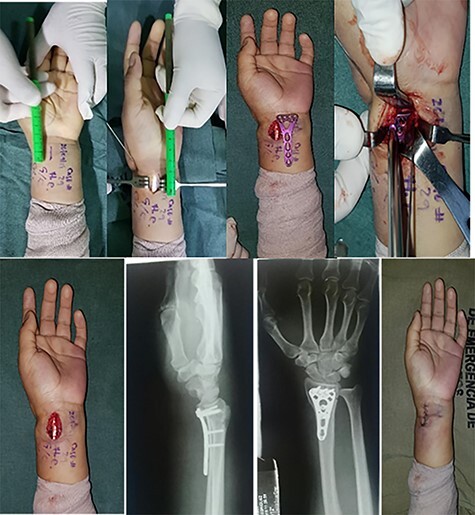
Longitudinal I shape incision. (**a**) Measurement of the incision. (**b**) The I incision. (**c**) The plate over the forearm. (**d**) Introducing the plate inside the forearm. (**e**) The forearm with the plate introduced. (**f**) Lateral X-ray of the wrist. (**g**) Anteroposterior X-ray of the wrist. (**h**) The incision already sutured.

As a first step the proximal wrist crease is located, 2 cm below and locating the FCR on its axis, a longitudinal 3-cm incision is made in the skin; after this a longitudinal incision is made on the fascia of the forearm; in this way the FPL and radial vessels can be separated (Fig. 2). Distal dissection is performed to locate the line (Watershed line) visualizing articular surface and palmar radiocarpal ligaments. The single incision allows use the elasticity of the skin and introduce the anatomical locking volar plate for distal radius plate and place the screws through a proximal and distal port. The reduction is facilitated by longitudinal dissection of a part of the PQ.

**Figure 3:**
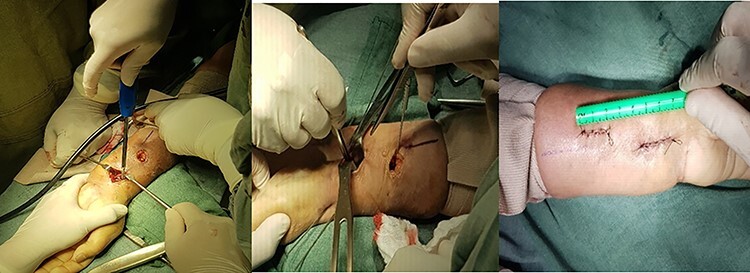
Double-incision longitudinal II. (**a**) Double incision with the plate already introduced in the forearm. (**b**) Verification of the osteosynthesis in the radius. (**c**) Both incisions already sutured

**Table 1:** Mayo clinical wrist evaluation

	Amount	Results (degrees)		Mayo wrist score			Complications	Delay fracture union
		F	E	Fair	Good	Excellent		
Incision T	10	74°	71°	3	4	3	2	1
Incision I	7	78°	78°	1	4	2	1	0
Incision II	9	78°	75°	3	3	3	1	0

**Table 2:** Mayo clinical wrist evaluation

	Amount	Results (degrees)		Mayo wrist score	Complications	Delay fracture union
		F	E			
Incision T	10	74°	71°	Good	2	1
Incision I	7	78°	78°	Good	1	0
Incision II	9	78°	75°	Good	1	0

**Table 3:** Mayo wrist score results (*n* = 26)

Total point score	Amount	Percentage
Excellent	8	30.77%
Good	11	42.31%
Fair	7	26.92%
Poor	0	0.00%

#### Double incision longitudinal II

The proximal wrist crease is located and 2 cm below it, on the axis of the FCR, the first distal incision of 2.5 cm is made and the other proximal incision of 2.5 cm longitudinal, with an average separation of 2.0 cm between one and the other (Fig. 3). A dissection is performed between the FCR that moves laterally and the radial vessels that move medially. The plate is inserted through the distal incision; a tunnel is made in the distal edge of the PQ, and the plate is slid in a proximal direction maintaining the reduction of the fracture; one or two 1.5-mm Kirschner wires are placed to temporarily fix the fracture, which goes diagonally from the radius styloid toward proximal, then proceeds to fix the plate with the previously described technique.

## DISCUSSION

It must be remembered that the objective of a smaller incision is to return to activity as soon as possible; therefore, at this point we can highlight that the success was achieved since we only had one delay in the healing of the wound (Table 4). Another 25 cases healed 10 days after the surgery, something that was very reassuring for the patients. After that it is important to see the functionality of the operated patients, and as the objective is to describe the techniques and to be able to apply them to people in general; the patients were chosen randomly, without preferences of any kind. To evaluate the functionality of the wrist, the Mayo Clinic wrist score was used (Tables 1–3), which gave us 31% excellent results, 42% good results, 27% fair results; with this we can highlight that the approaches are effective both in healing and in functionality. When evaluating the type of incision that was used with the patients, it was evident that the type of incision most widely used was type T, with an approximate flexion of 74° and extension of 71°. According to the classification of Mayo Clinic, a good result on most of the osteosynthesis was obtained; on the other hand, four complications were found; we found one delay in consolidation. Regarding the single incision, it presented an average flexion and extension result of 78°, with most patients in the Mayo classification with good results, presenting only one complication. Finally, the double incision showed a flexion result of 78° and an extension of 75°, with an even result in the Mayo classification as acceptable, good and excellent. It only presented one complication. Among the complications found were intra-articular screw, tendonitis of the FCR, neuropraxia of the median nerve and delay in healing the wound (Table 4). With this we can conclude that the approaches meet the criteria for which they were created, are functional, have better aesthetics and less recovery time, in addition to being applicable to anyone.

**Table 4:** Complications (*n* = 26)

Type	Amount	Percentage
Intra-articular screw	1	3.85%
Tendinitis of the FCR	1	3.85%
Neuropraxia of the median nerve	1	3.85%
Delayed wound healing	1	3.85%
None	22	84.62%

## CONFLICT OF INTEREST STATEMENT

None declared.
